# Comparative Evaluation of Tear Strength of Two Platinum-Based Maxillofacial Silicone Materials: Siloczest LSR With and Without the Addition of 2.5% Zinc Oxide (ZnO) Nanoparticles and Technovent (m511)—An In Vitro Study

**DOI:** 10.1155/tswj/3581428

**Published:** 2025-10-17

**Authors:** Smitha Sharan, D. L. Sarandha, S. Sujana, Mehul A. Shah, Vignesh Kamath

**Affiliations:** ^1^Department of Prosthodontics Including Crown and Bridge, Dayananda Sagar College of Dental Sciences and Hospital, Bengaluru, India; ^2^Department of Public Health Dentistry, Government Dental College and Hospital, Jamnagar, Gujarat, India; ^3^Department of Prosthodontics Crown and Bridge, Manipal College of Dental Sciences Mangalore, Manipal Academy of Higher Education, Manipal, Karnataka, India

**Keywords:** maxillofacial silicone material, nanoparticles, tear strength, zinc oxide

## Abstract

**Objective:**

The present study aims to evaluate and compare the tear strength of an indigenous platinum-based maxillofacial silicone material (Siloczest LSR [Liquid Silicone Rubber]) with and without the incorporation of 2%–2.5% zinc oxide nanoparticles, using an imported platinum-based silicone material (Technovent M511) as the benchmark control.

**Methodology:**

Sixty-six unnicked 90° angle specimens (ASTM D624 [American Society for Material and Testing)) were fabricated and divided into three groups: Group A—Siloczest LSR without zinc oxide nanoparticles; Group B—Siloczest LSR with 2.5% zinc oxide nanoparticles; and Group C (control)—Technovent M511. Tear strength was evaluated using a Universal Testing Machine (UTM), and results were statistically analysed to compare the performance of the materials.

**Results:**

Siloczest LSR showed significantly higher tear strength (22.64 N/mm) than Technovent (10.08 N/mm, *p* < 0.001). The addition of 2.5% ZnO nanoparticles reduced tear strength to 20.54 N/mm (*p* < 0.05), indicating no reinforcement benefit.

**Conclusion:**

Siloczest LSR, an indigenous platinum-based maxillofacial silicone, exhibited higher tear strength than the imported Technovent material, supporting its potential use in clinical prosthetic applications as a cost-effective maxillofacial material. However, the addition of 2.5% zinc oxide nanoparticles did not improve tear strength and led to a slight reduction. These results highlight the need for further investigation into nanoparticle concentration and dispersion methods to optimize reinforcement without compromising the material's mechanical integrity and clinical validation.

## 1. Introduction

Maxillofacial deformities and defects can lead to significant psychosocial distress, affecting both the physical and emotional well-being of patients. The rehabilitation of such defects using maxillofacial prostheses plays a crucial role in restoring function, aesthetics, and self-esteem [1]. Among the materials used for these prostheses, room-temperature vulcanizing (RTV) silicones—especially platinum-based systems—are favoured due to their biocompatibility, flexibility, and ease of colour matching. However, clinical experience shows that these materials often suffer from marginal tearing and mechanical degradation over time, compromising the prosthesis's longevity and effectiveness [2, 3].

To address these shortcomings, researchers have focused on enhancing the mechanical properties of silicone elastomers. Incorporation of nanoparticles, particularly zinc oxide (ZnO) nanoparticles, has emerged as a promising strategy due to their excellent physicochemical characteristics, such as small particle size, high surface-to-volume ratio, and strong interaction with silicone matrices. These properties facilitate better stress transfer and energy dissipation within the polymer, potentially improving tear resistance and durability [4–6].

Recent studies have provided encouraging data on ZnO nanoparticle reinforcement. Incorporating ZnO nanoparticles into RTV silicones significantly improved both tensile and tear strength, while maintaining acceptable elastic properties [7]. Increased tear strength and dimensional stability in nanoparticle-reinforced silicones used for facial prostheses, particularly under thermomechanical stress [7].

More recent in vitro studies provide mixed outcomes on ZnO nanoparticle reinforcement. Karaman and Altıntaş [8] observed significant improvements in tear strength with 2 wt% ZnO added to M511 platinum silicone (*p* < 0.05). While Abdalqadir et al. [9] reported tear strength gains using 3 wt% ZnO with sonication-aided dispersion (*p* < 0.05). In contrast, Khanna et al. [10] found that 2% ZnO significantly reduced tear strength (*p* = 0.014) in heat-vulcanized silicone, unlike TiO_2_ or PTFE fillers which enhanced it. Anjali and Chethan [11] also demonstrated that ZnO-reinforced silicone after 6 months of weathering did not outperform TiO_2_. A systematic review similarly noted tear strength gains at 2–2.5 wt% nano-oxides but performance decline at higher loadings due to particle aggregation [12]. These results emphasize the need for precise optimization of ZnO concentration and dispersion methods—consistent with our findings that 2.5% ZnO failed to reinforce tear strength.

Despite these advancements, there remains a gap in the literature regarding indigenous silicone materials, particularly in the Indian context. Siloczest LSR, a recently introduced Indian-manufactured platinum-cured maxillofacial silicone, is marketed as a cost-effective alternative to established imported brands such as Technovent M511. However, there is limited published research evaluating its mechanical properties or its behaviour when modified with reinforcing agents like ZnO nanoparticles.

Therefore, the present study aims to compare the tear strength of Siloczest LSR with and without the addition of 2.5% ZnO nanoparticles and to benchmark it against the imported Technovent M511. Despite the increasing availability of indigenous silicone materials such as Siloczest LSR, there is a notable lack of published data validating their mechanical properties, particularly tear strength, which is essential for the clinical longevity of maxillofacial prostheses. Moreover, while zinc oxide (ZnO) nanoparticles have demonstrated mechanical reinforcement effects in various polymer systems, their specific influence on Siloczest LSR has not been previously reported. This study aims to address this gap by evaluating and comparing the tear strength of Siloczest LSR with and without the addition of 2.5% ZnO nanoparticles, using the imported Technovent M511 platinum-based silicone as a benchmark. The null hypothesis proposed is that there is no statistically significant difference in tear strength among the three groups. Conversely, the alternative hypothesis states a statistically significant difference in tear strength across the groups.

## 2. Materials and Methods

**Figure 1:**
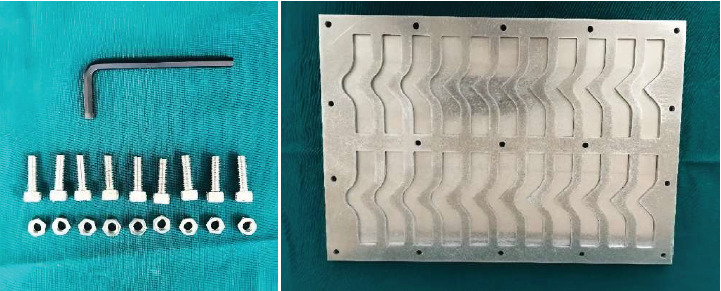
Metal mould used for specimen fabrication.

A total of 66 specimens were fabricated according to ASTM D624 standards for tear strength testing (Figure 1). The specimens were divided equally into three groups:

- Group A (test Group A): 22 specimens made from Siloczest LSR (platinum-based maxillofacial silicone) without ZnO nanoparticles.
- Group B (test Group B): 22 specimens made from Siloczest LSR with 2.5% ZnO nanoparticles.
- Group C (control group): 22 specimens made from Technovent M511 (platinum-based maxillofacial silicone).

**Figure 2:**
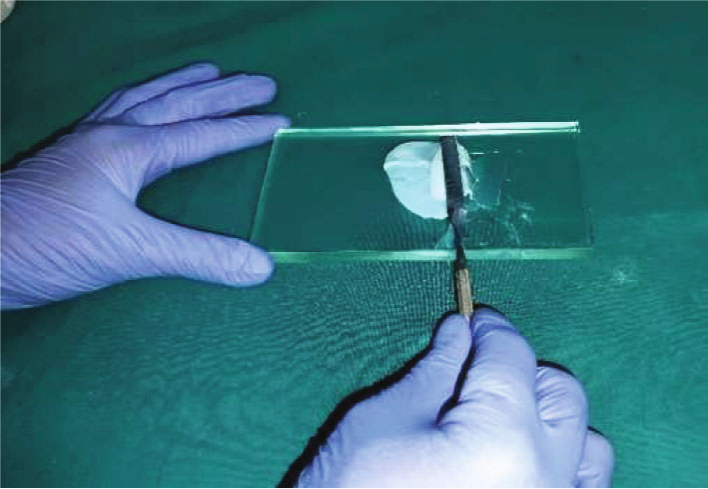
Mixing of premeasured maxillofacial silicone material using a metal spatula and a glass slab, in a sweeping motion to achieve a homogenous mixture.

**Figure 3:**
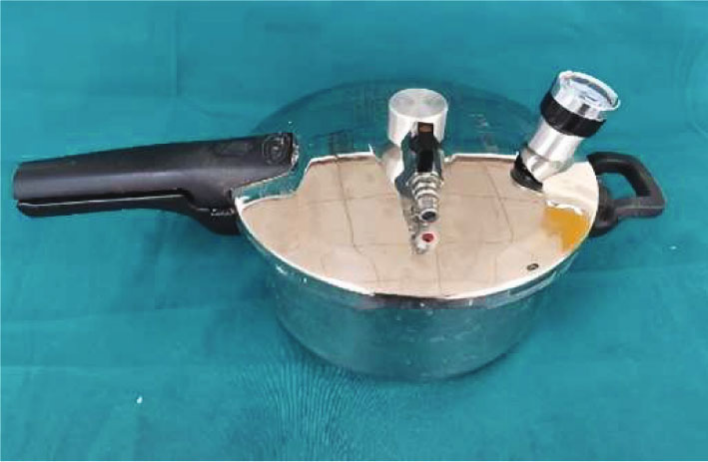
Pressure pot used to eliminate entrapped air bubbles during the curing of maxillofacial silicone specimens.

The base and catalyst were mixed in manufacturer-recommended ratios: 1:1 (Siloczest) and 10:1 (Technovent) by weight. Mixing was done in a sweeping motion on a glass slab using a metal spatula until a homogeneous consistency was achieved (Figure 2). The mix was then transferred to a pressure pot (Figure 3) to eliminate entrapped air.

ZnO nanoparticles were procured from Adnano Technologies Pvt. Ltd., Bengaluru, Karnataka, India. As per the manufacturer's technical datasheet, the particles had an average size of 40–60 nm (confirmed via TEM) and were spherical, uncoated, and >99% pure with no surface functionalization. Prior to mixing, the nanoparticles were dried at 80°C for 2 h to remove moisture. No chemical treatment or coating was performed.

To prepare Group B specimens, 2.5% by weight ZnO nanoparticles were gradually added to the Siloczest LSR base and manually pre-mixed. This was followed by mechanical stirring at 1000 rpm for 10 min using a laboratory stirrer to promote uniform dispersion. No ultrasonication was employed. The catalyst was then added, and the blend was poured into ASTM D624 moulds, followed by vacuum curing at room temperature for 24 h to enhance dimensional stability.

An exact concentration of 2.5% ZnO was chosen based on previous literature. Mahmood et al. [7] also noted that optimal reinforcement is below 2.5%, with higher concentrations leading to agglomeration and brittleness.

**Figure 4:**
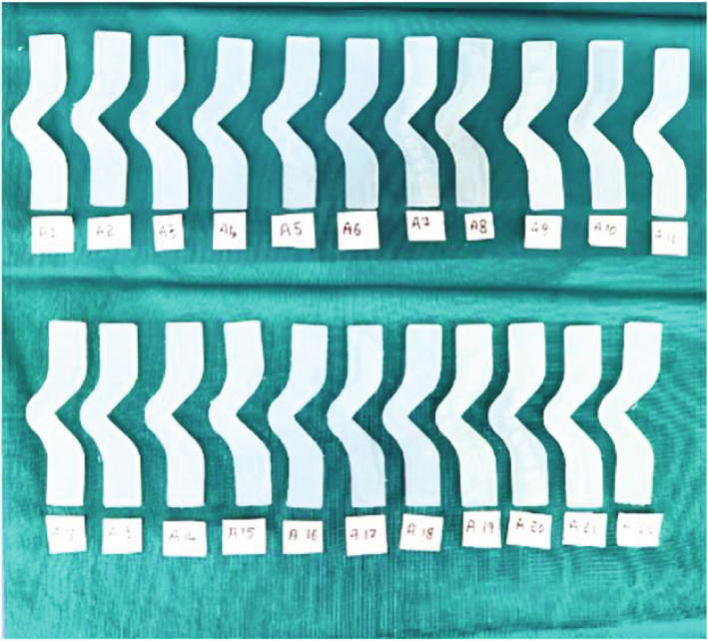
Group A—Siloczest LSR—platinum-based maxillofacial silicone material (Test Group 1).

**Figure 5:**
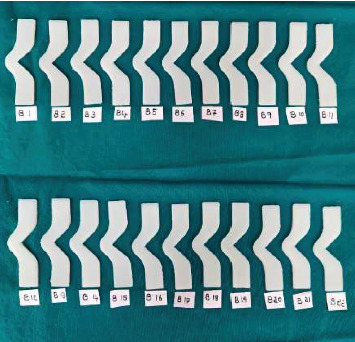
Group B—Siloczest LSR—platinum-based maxillofacial silicone material with addition of 2.5% zinc oxide (ZnO) nanoparticles (Test Group 2).

**Figure 6:**
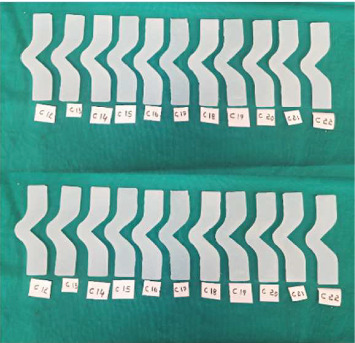
Group C—Technovent (M511)—platinum-based maxillofacial silicone material (control group).

All groups were processed under identical environmental and curing conditions to maintain comparability. After mixing, the materials were poured into standardized moulds and placed in a pressure pot at 2.5 bar (36 psi) for 30 min. Curing was performed at room temperature (23°C ± 2°C) and 50% ± 5% relative humidity for 24 h. No post-curing or heat treatment was conducted. After 7–8 h of polymerization, the specimens were carefully demoulded. Any excess material or flash was trimmed using a scalpel (Figures 4, 5, and 6). All specimens were inspected for dimensional accuracy before mechanical testing.

### 2.1. Evaluation of Tear Strength

**Figure 7:**
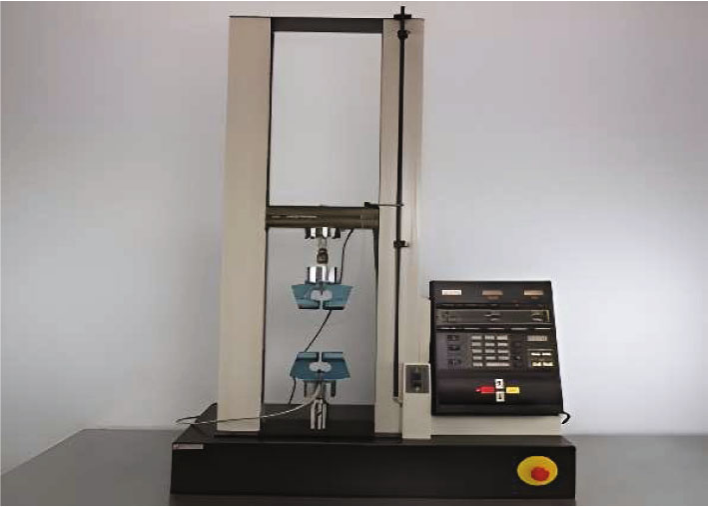
Universal Testing Machine (UTM) used to measure tear strength of maxillofacial silicone specimens according to ASTM D624 standards.

All the specimens were tested for the tearing power. Every specimen was placed in the Universal Testing Machine's jaws (UTM) (Figure 7), which applies a load on prefabricated samples as the jaws are separated. A steady increase in force takes place until reaching a point where the specimens break. A maximum jaw separation load of hundred Newton (N) [6] at the speed of 500 ± 50 mm/min (20 ± 2.0 in./min) was applied for the test specimens, and the test was terminated as the samples broke. The accuracy of the Universal Testing Machine was certified by annual calibration, and a quality check was done every 4 days to ensure reliable test results.

### 2.2. Statistical Analysis Design

The data obtained from the test were subjected to statistical analysis. The tear strength of the three groups was compared using a one-way ANOVA. A 95% confidence level and a significance threshold of *p* < 0.05 were established. To conduct intergroup comparisons between the test groups and control groups, post hoc Tukey's test was used afterward.

## 3. Results

The mean tear strength of Siloczest LSR platinum-based maxillofacial silicone material without zinc oxide nanoparticles was 22.64 N/mm, while the mean tear strength for Siloczest LSR with the addition of 2.5% zinc oxide nanoparticles was 20.54 N/mm. Both values were significantly higher than that of the imported Technovent platinum-based maxillofacial silicone material, which exhibited a mean tear strength of 10.08 N/mm. However, the addition of zinc oxide nanoparticles did not result in an increase in tear strength compared to the unmodified Siloczest material. On the contrary, a slight reduction in tear strength was observed with nanoparticle incorporation.

• Group I (Technovent): 10.08 ± 0.94 N/mm
• Group II (Siloczest without ZnO) had the highest mean tear strength: 22.64 ± 1.27 N/mm
• Group III (Siloczest with ZnO): 20.54 ± 1.31 N/mm

One-way analysis of variance (ANOVA) for tear strength was used to compare the experimental groups' data to the control group at a significance level of 0.05 for all tests within the statistical analysis (Table 1).

**Figure 8:**
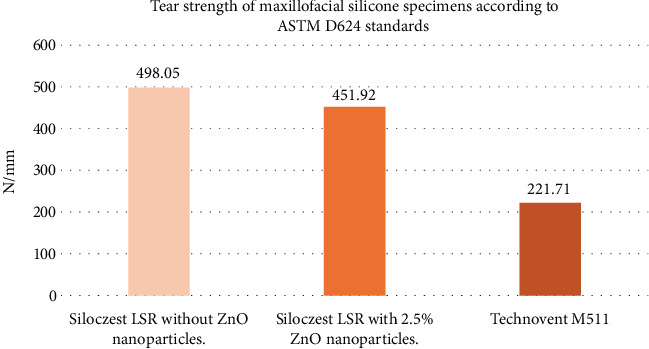
Summation of Group A value, Group B value and Group C value.

As a result of the F computed value exceeding the F table value, the alternative hypothesis is accepted and the null hypothesis is rejected. Given the rejection of the H_0, a post hoc Tukey's test is used. Multiple inter-comparisons were conducted in this test, and the results showed that there is a substantial difference between Group A and Group C, as well as between Group B and Group C (Figure 8).

## 4. Discussion

A prosthetic replacement made of a range of materials and procedures has been used to correct acquired surgical deformities since the sixteenth century. As part of the ablative technique, definitive reconstruction should be carried out whenever feasible. In numerous instances, head and neck defects can be brought back to almost normal function and appearance by the coordinated use of final repair and maxillofacial prosthetic therapy. To make such a prosthesis, a great deal of expertise and experience are needed. The choice of materials to be used in the prosthesis' construction will be essential to meeting all of these parameters [13].

This study aimed to compare the tear strength of Siloczest LSR (an indigenous platinum-based maxillofacial silicone) with and without 2.5% zinc oxide (ZnO) nanoparticles against Technovent M511, an imported benchmark material. The highest tear strength was observed in the unmodified Siloczest group (22.64 N/mm), followed by the ZnO-reinforced group (20.54 N/mm), and the lowest in Technovent (10.08 N/mm). Although both Siloczest groups outperformed Technovent, the addition of ZnO did not improve the tear strength. The source of variation calculated between the samples using one-way ANOVA table gave an F ratio of 71.63 and the sources of variation calculated within the sample gave a *p* value of 0.00001 which is clearly < 0.05. As a result, the test groups' (A and B) and the control group's (C) tear strengths differed significantly.

Previous studies, including by a study by Mahmood et al. [7], have reported significant improvements in the mechanical properties of maxillofacial silicones with the incorporation of ZnO nanoparticles, particularly at concentrations around 2.5%. These enhancements have been attributed to effective nanoparticle dispersion and interfacial bonding with the silicone matrix. Recent in vitro studies show mixed results on ZnO nanoparticle reinforcement. While Karaman and Altıntaş [8] and Abdalqadir et al. (2020/2022) reported significant improvements in tear strength at 2–3 wt% ZnO with proper dispersion (*p* < 0.05), Khanna et al. [10] found a significant reduction at 2% ZnO (*p* = 0.014). Anjali and Chethan [11] also noted inferior performance compared to TiO_2_ after weathering. A systematic review supports improved strength at 2%–2.5% but decline at higher levels due to agglomeration. These findings highlight the need for optimal ZnO concentration and dispersion, aligning with our observation that 2.5% ZnO reduced tear strength [12].

Contrary to expectations, the addition of 2.5% ZnO nanoparticles to Siloczest LSR resulted in a slight reduction in tear strength. This suggests that ZnO may not universally reinforce all silicone formulations and that its effect may be dependent on the base material's compatibility and dispersion quality.

The proper concentration of each pigment was selected previously based on the pilot studies. The test groups had the highest tear strength in comparison to the control group, and a statistically significant difference was seen between the two groups. The deviation of test results from those of the literature search regarding the enhancement of tear strength with the addition of zinc oxide nanoparticles [14, 15, 16, 17] creates a room to analyse the following:

a. Effect of nanoparticles on the indigenous silicone
b. Concentration of nanoparticles required to enhance the tear strength of indigenous silicone

The decreased performance observed with ZnO incorporation could be attributed to several factors:

- Agglomeration of nanoparticles due to the absence of ultrasonication, which could create stress concentration zones within the matrix
- Inadequate interfacial bonding between ZnO and the silicone due to the lack of surface treatment
- Differences in base polymer properties between Siloczest and those used in other studies
- Suboptimal mixing during mechanical stirring, potentially leading to uneven nanoparticle distribution

The results underscore the complexity of nanoparticle reinforcement and highlight that addition alone is not sufficient—dispersion, surface interaction, and matrix compatibility play crucial roles. These findings warrant a more nuanced interpretation rather than assuming ZnO universally improves tear strength.

While many studies confirm the positive role of ZnO nanoparticles in silicone reinforcement, our findings align with some recent investigations that report diminished or neutral effects when nanoparticles are not adequately dispersed or tailored to the specific polymer system. For example, studies by Nafea et al. and Yilmaz et al. caution that poor mixing techniques or nanoparticle agglomeration can negate expected benefits [14, 15].

This study provides valuable insight into the limitations of applying generalized conclusions across different silicone formulations and emphasizes the need for tailored optimization of nanoparticle concentration and incorporation techniques.

### 4.1. Implications of the Findings

The results of this study indicate that the indigenous Siloczest LSR silicone material demonstrates superior tear strength compared to the widely used imported Technovent material, suggesting its potential as a cost-effective alternative for maxillofacial prosthetic applications. However, the incorporation of 2.5% zinc oxide nanoparticles did not improve the mechanical performance and instead resulted in a statistically significant reduction in tear strength. These findings underscore the importance of optimizing nanoparticle concentrations and dispersion methods before clinical translation. While promising, further in vivo validation and long-term performance evaluations are warranted before recommending the material for routine clinical use.

### 4.2. Limitations of the Study

This in vitro study has several limitations that may affect its clinical applicability. First, nanoparticle characterization was not performed, making it difficult to confirm dispersion or interfacial bonding. Second, only a single ZnO concentration (2.5%) was evaluated, limiting insights into dose-dependent effects. Third, advanced analytical techniques such as SEM, EDS or FTIR were not employed, restricting the understanding of nanoparticle distribution and chemical interaction with the silicone matrix. Fourth, no aging or weathering protocols were applied, so long-term performance under clinical conditions remains unknown. Additionally, only RTV silicones were tested, excluding HTV materials that may behave differently. Manual mixing and curing may have introduced internal porosity, potentially impacting mechanical properties.

### 4.3. Future Scope

Future research should employ advanced characterization tools (SEM, etc.) to validate nanoparticle dispersion. Testing multiple concentrations and improved dispersion methods (e.g., ultrasonication) could refine mechanical outcomes. Aging and weathering simulations are essential for long-term durability assessment. Including HTV silicones and clinically relevant geometries will improve translational relevance.

### 4.4. Clinical Significance of the Study

It is observed that over the time of repeated usage of the prosthesis the edges of maxillofacial silicone material will be torn. Hence, it is necessary to enhance the tear strength of the maxillofacial silicone material for better longevity of the prosthesis.

Apart from improving the mechanical properties the maxillofacial silicone materials should be cost effective. Especially in a middle-income country like India, it is necessary that the maxillofacial silicone material should be readily available to treat various conditions which result in loss of the facial structures due to cancer, road traffic accident, congenital defects, and many more. Using indigenous maxillofacial silicone materials will reduce the treatment cost for the patients, as the imported maxillofacial are expensive adding additional treatment cost for the patients.

## 5. Conclusion

This in vitro study evaluated the tear strength of an indigenous maxillofacial silicone material (Siloczest LSR) with and without the addition of 2.5% zinc oxide nanoparticles, using Technovent (M511) as the control. The unmodified Siloczest LSR demonstrated the highest tear strength (22.64 N/mm), followed by the ZnO-reinforced group (20.54 N/mm), and Technovent (10.08 N/mm). The addition of ZnO nanoparticles did not enhance tear strength compared to the unmodified Siloczest, highlighting the importance of optimal nanoparticle dispersion and concentration.

While the indigenous material showed promising mechanical performance, the study was limited by the absence of nanoparticle characterization, aging simulation, and clinical geometry. Future investigations should explore multiple concentrations, advanced characterization techniques, and long-term durability to better translate in vitro findings into clinical applications.

## Figures and Tables

**Table 1:** Calculations for one-way ANOVA.

**Sources of variation**	**Sum of squares**	**Degrees of freedom**	**Mean squares**	**F** **ratio**
Between the sample	SSC = 1991.44	V = C -1 = 3-1 = 2	MSC = SSC/V = 1991.44/2 = 995.72	MSC/MSE = 995.72/13.90 = 71.63
Within the sample	SSE = 876	V = n – c = 22⁣^∗^3 – 3 = 63	MSE = SSE/V = 876/63 = 13.90	*p* value = 0.00001(<0.05)

## Data Availability

All data used/analysed in this paper are available with the corresponding author and will be shared upon reasonable request.
